# Genomic prediction across years in a maize doubled haploid breeding program to accelerate early-stage testcross testing

**DOI:** 10.1007/s00122-020-03638-5

**Published:** 2020-06-30

**Authors:** Nan Wang, Hui Wang, Ao Zhang, Yubo Liu, Diansi Yu, Zhuanfang Hao, Dan Ilut, Jeffrey C. Glaubitz, Yanxin Gao, Elizabeth Jones, Michael Olsen, Xinhai Li, Felix San Vicente, Boddupalli M. Prasanna, Jose Crossa, Paulino Pérez-Rodríguez, Xuecai Zhang

**Affiliations:** 1 Institute of Crop Sciences, Chinese Academy of Agricultural Sciences, Beijing, China; 2 International Maize and Wheat Improvement Center (CIMMYT), Texcoco, Mexico; 3 CIMMYT-China Specialty Maize Research Center, Shanghai Academy of Agricultural Sciences, Shanghai, China; 4 Crop Breeding and Cultivation Research Institute, Shanghai Academy of Agricultural Sciences, Shanghai, China; 5 College of Bioscience and Biotechnology, Shenyang Agricultural University, Shenyang, Liaoning China; 6 Plant Breeding and Genetics Section, School of Integrative Plant Science, Cornell University, Ithaca, NY USA; 7 Institute of Biotechnology, Cornell University, Ithaca, NY USA; 8 International Maize and Wheat Improvement Center (CIMMYT), P. O. Box 1041, Nairobi, Kenya; 9 Colegio de Postgraduados, Texcoco, Estado De México Mexico

## Abstract

****Key message**:**

**Genomic selection with a multiple-year training population dataset could accelerate early-stage testcross testing by skipping the first-stage yield testing, which significantly saves the time and cost of early-stage testcross testing.**

**Abstract:**

With the development of doubled haploid (DH) technology, the main task for a maize breeder is to estimate the breeding values of thousands of DH lines annually. In early-stage testcross testing, genomic selection (GS) offers the opportunity of replacing expensive multiple-environment phenotyping and phenotypic selection with lower-cost genotyping and genomic estimated breeding value (GEBV)-based selection. In the present study, a total of 1528 maize DH lines, phenotyped in multiple-environment trials in three consecutive years and genotyped with a low-cost per-sample genotyping platform of rAmpSeq, were used to explore how to implement GS to accelerate early-stage testcross testing. Results showed that the average prediction accuracy estimated from the cross-validation schemes was above 0.60 across all the scenarios. The average prediction accuracies estimated from the independent validation schemes ranged from 0.23 to 0.32 across all the scenarios, when the one-year datasets were used as training population (TRN) to predict the other year data as testing population (TST). The average prediction accuracies increased to a range from 0.31 to 0.42 across all the scenarios, when the two-years datasets were used as TRN. The prediction accuracies increased to a range from 0.50 to 0.56, when the TRN consisted of two-years of breeding data and 50% of third year’s data converted from TST to TRN. This information showed that GS with a multiple-year TRN set offers the opportunity to accelerate early-stage testcross testing by skipping the first-stage yield testing, which significantly saves the time and cost of early-stage testcross testing.

## Introduction

Modern breeding tools and technologies, such as doubled haploid (DH) technology and genomic selection (GS), provide new approaches to increase the genetic gain in plant breeding. The DH technology, firstly proposed in maize breeding more than half a century ago, allows breeders to obtain homozygous inbred lines in two generations compared to more than six generations of inbreeding in conventional breeding (Prasanna 2012; Sleper and Bernardo 2016). The other advantage of DH technology is the selection effectiveness, due to the genetic uniformity of the tested genotypes across seasons (Masuka et al. 2017). As the cost of developing DH lines decreased, thousands of DH lines are able to be generated in a maize breeding program every year. Therefore, the main task for a maize breeder is to estimate the breeding values of thousands of DH lines, rather than to generate thousands of homozygous inbred lines annually.

GS is a molecular marker-based selection method, in which the marker effects are estimated in the training population (TRN) based on prior phenotypic and molecular marker data, and then the marker effects estimated from the training population (TRN) are used to predict the genomic estimated breeding value (GEBV) of the genotypes in a target breeding population, which have been genotyped but not phenotyped (Meuwissen et al. 2001). In maize, GS has been implemented in several studies in various kinds of genetic and breeding populations to estimate the genomic prediction accuracy and evaluate the genetic gain (Crossa et al. 2014; Beyene et al. 2015; Zhang et al. 2017a and b). The main factors affecting genomic prediction accuracy include the size of TRN, the relationship between TRN and TST (testing population), the genetic architecture and the heritability of the target trait, the genotype by environment interaction, statistical models, etc. (Beyene et al. 2015;  Guo et al. 2014; Kadam et al. 2016; Kadam and Lorenz 2018). Brandariz and Bernardo (2019) showed that the relationship between TRN and TST was more important in improving prediction accuracy than the size of TRN. Multiple-environment trials have an important role in plant breeding to assess the genotype by environment interaction and select breeding materials with good preference and stability. However, most previous GS studies only used single-environment prediction models. Until recently, several studies showed that incorporating genotype by environment interaction into the statistical models was able to improve the genomic prediction accuracy (Burgueño et al. 2012; Jarquín et al. 2014; Sousa et al. 2017; Zhang et al. 2015).

The breeding data in a maize breeding program are dynamic and complex. As part of the routine maize product development pipeline, thousands of DH lines derived from genetically diverse parents and populations are able to be generated for each breeder every year at an affordable cost; the general combining ability and breeding value of this large number of DH lines need to be evaluated in the first-stage yield testing trials, i.e., the early-stage testcross testing, where the testcross formed between a large number of DH lines and a few testers is always phenotyped in multiple-environment trials. Several different testers from the complementary heterotic groups are used to make the testcross, according to the genetic background of the DH lines (Albrecht et al. 2011 and 2014). In a maize breeding program, the process of early-stage testcross testing repeats every year, and the DH lines tested across years are partially connected as full-sibs or half-sibs, as the key inbred lines are repeated as parental lines for recycling for several years (Schrag et al. 2018; Rio et al. 2019). GS enables the GEBV estimation and selection on the untested DH lines prior to phenotyping (Andorf et al. 2019; Brauner et al. 2018). In early-stage testcross testing, GS offers the opportunity of replacing expensive multiple-environment phenotyping and phenotype-based breeding value selection with lower-cost genotyping and GEBVs-based selection. However, the strategy of implementing GS to replace phenotyping in the early-stage testcross testing needs to be further explored by employing the multiple years of breeding data, due to the complexity of the early-stage testcross testing  (Marulanda et al. 2016).

In the present study, a total of 1528 DH lines, phenotyped in multiple-environment trials in three consecutive years and genotyped with a low-cost per-sample genotyping platform of rAmpSeq, were used to explore how to implement GS to accelerate the early-stage testcross testing in a maize doubled haploid breeding program. rAmpSeq is a newly developed sequencing method, which scores thousands of markers with the cost of less than 5 US dollars per sample (Buckler et al. 2016). The main objectives of the present study are to: (1) estimate the genomic prediction accuracies in the within and across year analyses; (2) evaluate the effect of genomic prediction model incorporating genotype by environment interaction on the genomic prediction accuracy estimation; (3) explore the breeding strategy of implementing GS to accelerate the early-stage testcross testing in a maize doubled haploid breeding program.

## Materials and methods

### Plant materials and field experiments

**Fig. 1:**
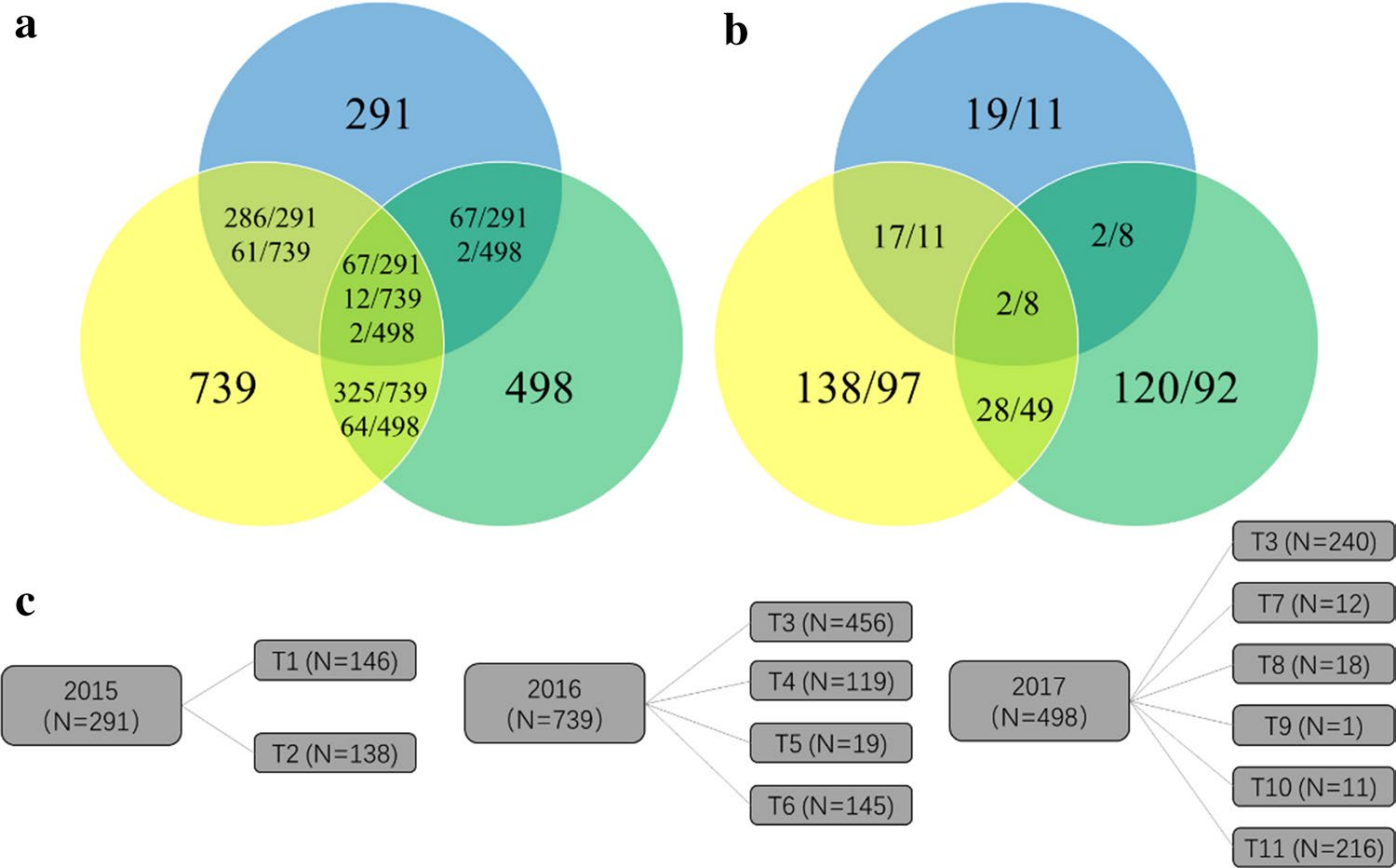
The basic information of the phenotypic dataset from three consecutive years from 2015 to 2017. **a** the number of full-sib and half-sib DH lines shared between different years; blue color—2015, yellow color—2016, green color—2017; **b** the number of biparental populations and the number of parental lines used to form biparental populations; blue color—2015, yellow color—2016, green color—2017; **c** number of testers used in each year and number of testcrosses evaluated in each tester

In the present study, a total of 1528 DH lines, developed by the lowland tropical maize breeding program of CIMMYT in Mexico in three consecutive years, were used to explore how to implement genomic prediction to accelerate early-stage testcross testing in a DH breeding program. In 2015, 291 DH lines from 19 biparental populations were derived from the F_1_ crosses made between 11 parental lines; the number of DH lines per population ranged from 1 to 53, with an average of 15. In 2016, 739 DH lines from 138 biparental populations were derived from the F_1_ crosses made between 97 parental lines; the number of DH lines per population ranged from 1 to 66, with an average of five. In 2017, 498 DH lines from 120 biparental populations were derived from the F_1_ crosses made between 92 parental lines; the number of DH lines per population ranged from 1 to 58, with an average of four (Fig. 1). The number of full-sib and half-sib DH lines shared between different years is shown in Fig. 1a. Among the 291 DH lines tested in 2015, 286 and 67 lines are full- or half-sibs with part of the 739 lines tested in 2016 and part of the 498 lines tested in 2017, respectively. Among the 739 DH lines tested in 2016, 61 and 325 lines are full- or half-sibs with the part of the 291 lines tested in 2015 and part of the 498 lines tested in 2017, respectively. Among the 498 DH lines tested in 2017, 2 and 64 lines are full- or half-sibs with part of the 291 lines tested in 2015 and part of the 498 lines tested in 2017, respectively. The number of full-sibs across the three tested years is 67, 12, and 2 in 2015, 2016, and 2017, respectively. The number of biparental populations and the number of parental lines used to form biparental populations are shown in Fig. 1b, as well as the number of biparental populations and the number of parental lines shared between different years. The number of evaluated biparental populations is 19, 138, and 120 in 2015, 2016, and 2017, respectively. The number of parental lines used to form biparental populations is 11, 97, and 92 in 2015, 2016, and 2017, respectively. The number of shared biparental populations is 17, 28, and 2 for the pairwise years of 2015 and 2016, 2016 and 2017, and 2015 and 2017, respectively. The number of shared parental lines is 11, 49, and 8 for the pairwise years of 2015 and 2016, 2016 and 2017, and 2015 and 2017, respectively. Across the three tested years, the number of shared biparental populations and the number of shared parental lines are 2 and 8, respectively.

The testcrosses made between each DH line and the corresponding tester from the complementary heterotic group were evaluated in multiple-environment trials for phenotypic data collection; the target trait of the present study was grain yield (GY). Each DH line only crossed with one tester to make the testcross, and the number of testers used for making testcross in the year of 2015, 2016, and 2017 was two, four, and six, respectively (Fig. 1c). The testers were different between years, only one tester, i.e., T3, appeared in both 2016 and 2017. The number of testcrosses made with each tester ranged from 1 to 456 across the 3 years. In 2015 and 2016, the testcrosses were phenotyped in Mexico in three experimental stations, i.e., Agua Fria (AF, 20°27′N, 97°38′W), Cotaxtla (Cot, 19°15′N, 96°12′W), and Tlatizapan (TL, 18°41′N, 99°07′W). In 2017, the testcrosses were only evaluated in two experimental stations in Agua Fria and Tlatizapan. The number of trials conducted in the year of 2015, 2016, and 2017 was five, seven, and seven, respectively. A different subset of DH lines was evaluated in each trial. Each trial was laid out in an *α*-lattice design with two replications, and one-row plot was planted with 5 m long, 0.75 m between rows, and 0.25 m between hills. For each trial, the best linear unbiased predictor (BLUP) values and broad-sense heritability (*H* ^2^) of GY were calculated in within and across location analyses using the META-R software (http://hdl.handle.net/11529/10201). The broad-sense heritability (coefficient of repeatability) equation for within environment is:

$$H^{2} = \frac{{\sigma_{g}^{2} }}{{\sigma_{g}^{2} + \sigma_{\varepsilon }^{2} /r}}$$

For the across locations analyses, broad-sense heritability equation is

$$H^{2} = \frac{{\sigma_{g}^{2} }}{{\sigma_{g}^{2} + \sigma_{gE}^{2} /n + \sigma_{\varepsilon }^{2} /nr}}$$

where $\sigma_{g}^{2}$ is the genotypic variance, $\sigma_{gE}^{2}$ is the genotype × environment variance, $\sigma_{\varepsilon }^{2}$ is the error variance, *n* is the number of environments, and *r* is the number of replications.

## Genotyping and genotypic data

The genomic DNA of all the DH lines was sent to Cornell University Biotechnology Resource Center (Ithaca, NY, USA) for repeat Amplification Sequencing (rAmpSeq). The details of the protocol have been described by Buckler et al. (2016), where the DNA library was constructed in 3072-plex and sequenced on Illumina HiSeq 2000, each sequence tag was treated as a unique dominant marker, the tags from the B73 reference genome were considered as the present markers; otherwise, the sequence tags not mapped to the B73 reference genome were considered as the absent markers. Initially, 7595 dominant markers identified from the intergenic regions were called for all the genotyped DH lines. The markers with minor allele frequency (MAF) less than 0.05 were discarded, resulting in 6137 markers for further analysis.

## Genomic prediction analyses

All the genomic prediction analyses were conducted using the BGLR library (Perez and de los Campos 2014) in R program version 3.6.1 (R Core Team 2019). A total of 300,000 MCMC (Markov chain Monte Carlo) samples were collected, with 250,000 discarded as burn-in. Thinning was done by keeping one of every 10 samples.

Both the single-environment (year–location combination) model (SM) and multiple-environment model (MM) were implemented to evaluate the effect of modeling the genotype by environment interaction into genomic prediction. In SM, let $y_{ij}$ be the GY testcross performance for genotype $j$, which was tested in the environment $i$. The GY testcross performance can be predicted as follows:

$$y_{ij} = \mu_{i} + g_{j} + e_{ij},$$

where $\mu_{i}$ is an intercept particular to a given environment, since we are fitting the model for each environment, and $E_{i}$ is the same for all individuals (a constant); therefore, we can reparametrize the model by writing $\mu_{i} = \mu + E_{i}$, $g_{j}$ is the random effect of the genotype *j*, and $e_{ij}$ is the model residuals with $e_{ij} \sim NI\left( {0,\sigma_{ei}^{2} } \right)$, where “*NI*” stands for normal and independent, and $\sigma_{{e_{i} }}^{2}$ is the variance for the residual in the environment *i* (stratified analysis model in López-Cruz et al. 2015). We assume that $\varvec{g} \sim MN\left( {\mathbf{0},\varvec{G}\sigma_{g}^{2} } \right)$, where **g** is a vector of $g_{j}$, *MN* stands for a multivariate normal distribution, $\sigma_{g}^{2}$ is the variance associated with genotypes, and $\varvec{G}$ is a genomic relationship matrix derived from marker scores. $\varvec{G} = {\varvec{ZZ}^{\prime}}/p$ with $\varvec{Z}$ being the matrix of markers centered and standardized (López-Cruz et al. 2015) and $p$ is the number of markers.

In MM, the SM is extended to include the main effect of environment (year–location combination) and the interaction between genotype and environment. Here, we described the model proposed by Jarquín et al. (2014) to include the interaction through the reaction norm model, as follows:

$$y_{ij} = \mu + E_{i} + g_{j} + gE_{ij} + e_{ij} ,$$

where $E_{i}$ is the effect of the environment, with $E_{i} \sim NIID\left( {0,\sigma_{E}^{2} } \right)$, and $gE_{ij}$ is the interaction between environment $i$ and genotype $j$. We assume $\varvec{gE} \sim MN( {\mathbf{0},[\varvec{Z}_{g} \varvec{GZ}_{g}^{'} } ]\# \varvec{Z}_{E} \varvec{Z}_{E} '\sigma_{gE}^{2} )$, where $\varvec{ gE}$ is a random term that represents the interaction between genotype and environment jointly, $\varvec{Z}_{g}$ is the design matrix that connects the phenotypes with genotypes, $\varvec{Z}_{E}$ is the design matrix for environments, and $\#$ is the Hadamard product (cell by cell) between two matrixes, and $\sigma_{gE}^{2}$ is the variance component associated with this term.

Two validation schemes were used to assess the accuracy of prediction models, where the prediction accuracy (r_MG_) was defined as the Pearson correlation between the observed and predicted phenotypes. A fivefold cross-validation scheme with 50 replications was used to generate the TRN and TST sets and assess the prediction accuracy. In each of the 50 replications, the observations in randomly selected fourfold were assigned as TRN, and the remaining observations in the rest fold were assigned as TST. In order to assess the prediction accuracy across years, an independent validation scheme was applied, where the TRN and TST were from different years, and TRN was either from 1 year of breeding data or from 2 years of breeding data.

The TRN using 2 years of breeding data always has bigger population size and larger environmental variation than using 1 year of data as TRN. For separating the effects of population size and environmental variation on estimation of the prediction accuracy, the random sampling selection was applied on the TRN using 2 years of breeding data, and the size of the TRN using 2 years of breeding data was adjusted same as the size of the TRN using 1 year of data. The 2 years of breeding data with adjusted size were used as the TRN set to predict the third year’s data as the TST set. The random sampling selection was repeated 30 times, SM was applied in the within and across location analyses, and the average prediction accuracy was estimated in the TST set.

## Results

### Phenotypic data analysis and heritability

The mean performance and broad-sense heritability of GY in each year–location combination are shown in Table 1. For each year–location combination, the average GY varied. In AF, the highest GY with 8.40 t/ha was observed in 2017 and the lowest GY with 7.01 t/ha was observed in 2015. In TL, the highest GY with 11.22 t/ha was observed in 2015 and the lowest GY with 9.19 t/ha was observed in 2016. Across all 3 years, the highest GY value was observed in TL and the lowest GY value was observed either in AF or in Cot. The average variation across years was 1.16 t/ha, which was smaller than the variation across locations of 2.56 t/ha, indicating that GY varied both across locations and across years.

**Table 1:** The basic information of the phenotypic dataset from three consecutive years from 2015 to 2017, including the number of trials evaluated in each year, mean value and standard error of the target trait grain yield (GY) evaluated in each location and each year, and the broad-sense heritability (*H* ^2^) estimated from the trials evaluated in each location and each year

Year	No. of trials	GY				*H* ^*2*^			
		AF	Cot	TL	Across locations	AF	Cot	TL	Across locations
2015	5	7.01 ± 0.88	4.57 ± 1.50	11.22 ± 1.68	7.60 ± 0.95	0.30–0.59	0.10–0.70	0.55–0.78	0.11–0.70
2016	7	7.51 ± 1.46	7.48 ± 1.40	9.19 ± 1.99	7.73 ± 1.25	0.56–0.92	0.27–0.86	0.57–0.88	0.39–0.92
2017	7	8.40 ± 1.44	–	9.56 ± 1.54	8.40 ± 1.14	0.01–0.85	–	0.01–0.78	0.01–0.73

*AF* Agua Fria; *Cot* Cotaxtla; *TL* Tlatizapan

The heritabilities of GY in most of the trials were moderate to high, only except for two trials evaluated in 2017, in which the heritabilities were lower than 0.05 (Table 1). The average heritability across locations and trials was 0.53, 0.71, and 0.39 in the years 2015, 2016, and 2017, respectively.

## Prediction accuracies estimated from the fivefold cross-validation schemes

**Fig. 2:**
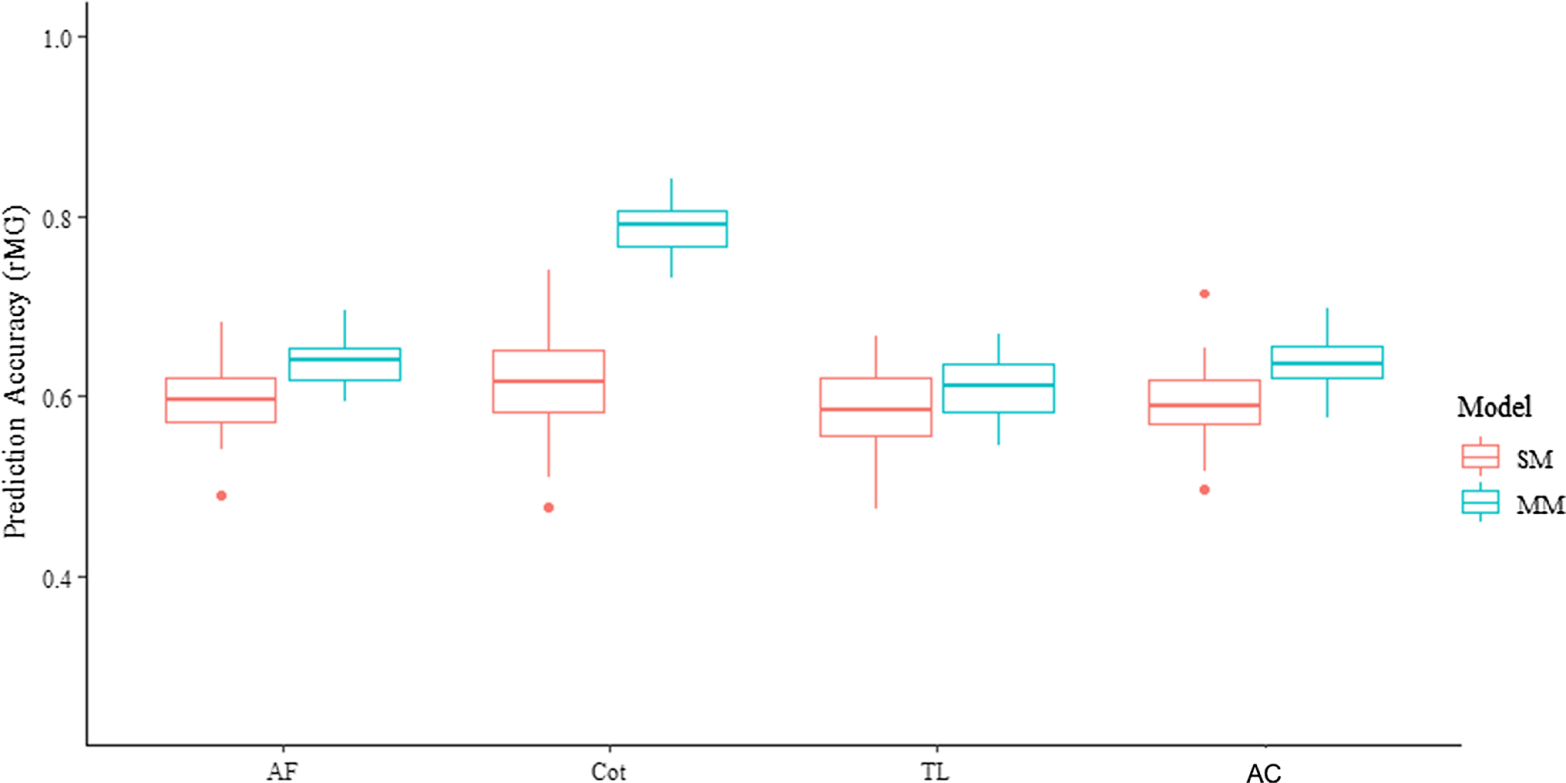
Prediction accuracies of grain yield (GY) estimated from the fivefold cross-validation schemes in the within and across location analyses using the single-environment model (SM) and multiple-environment model (MM). Within location analyses are from locations of Agua Fria (AF), Cotaxtla (Cot), and Tlatizapan (TL). AC is the across location analyses

The prediction accuracies estimated from the fivefold cross-validation schemes are shown in Fig. 2, when the SM and MM models were applied in the within location analyses and the across location analyses (Fig. 2). In either the within location analyses or the across location analyses, the prediction accuracies estimated from the MM were higher than those estimated from the SM. In the SM, the average prediction accuracy (r_MG_) was 0.60 across the within location analyses and the across location analyses. In the MM, the average prediction accuracy was 0.68 across the within location analyses and the across location analyses. Among all the three locations, the highest prediction accuracy was observed in Cot for SM and MM, where the highest heritability was observed.

## Prediction accuracies estimated from the independent validation schemes

The prediction accuracies estimated from the independent validation schemes are shown in Table 2. The SM and MM were applied in the within and across location analyses, the prediction accuracies were estimated, when the TRN and TST were from different years, and either 1 year of data or 2 years of data were used as TRN. When the 1-year data were used as TRN to predict the other year data as TST, the prediction accuracies of SM had an average value of 0.23 and ranged from − 0.11 to 0.36 in the within location analyses, and the prediction accuracies of MM in the within location analyses also had an average value of 0.23 and ranged from 0.10 to 0.36. In the across location analyses, the prediction accuracies of SM had an average value of 0.31 and ranged from 0.22 to 0.42, and the prediction accuracies of MM had an average value of 0.32 and ranged from 0.18 to 0.43. The prediction accuracies estimated from the across location analyses were higher than those estimated from the within location analyses, but the prediction accuracies estimated from the SM were similar to those estimated from the MM, despite in the within location analyses or in the across location analyses.

**Table 2:** The prediction accuracies estimated from the independent validation schemes either using 1-year data or 2-year data as TRN, when the single-environment model (SM) and multiple-environment model (MM) were applied in the within and across location analyses, and the TRN and TST were from different years

Location	Year in TST	Year in TRN (SM)				Year in TRN (MM)			
		2015	2016	2017	2 years	2015	2016	2017	2 years
AF	2015		0.36	0.26	0.42		0.36	0.26	0.43
	2016	0.19		0.15	0.22	0.19		0.14	0.18
	2017	0.27	0.32		0.35	0.28	0.31		0.34
Cot	2015		0.29				0.29		
	2016	− 0.11				0.13			
	2017								
TL	2015		0.27	0.10	0.26		0.17	0.10	0.36
	2016	0.34		0.35	0.40	0.21		0.35	0.35
	2017	0.15	0.25		0.23	0.17	0.25		0.25
Across locations	2015		0.48	0.19	0.50		0.48	0.22	0.49
	2016	0.32		0.35	0.36	0.34		0.35	0.39
	2017	0.26	0.35		0.38	0.27	0.34		0.38

*TRN* training set; *TST* testing set.

*AF* Agua Fria, *Cot* Cotaxtla, *TL* Tlatizapan

When the 2 years of data were used as TRN to predict the other year data as TST, the prediction accuracies of SM had an average value of 0.31 and ranged from 0.22 to 0.42 in the within location analyses, and the prediction accuracies of MM in the within location analyses had an average value of 0.34 and ranged from 0.18 to 0.43 (Table 2). In the across location analyses, the prediction accuracies of SM had an average value of 0.41 and ranged from 0.36 to 0.50, and the prediction accuracies of MM had an average value of 0.42 and ranged from 0.38 to 0.49 (Table 2).

When the 2 years of data were used as TRN to predict the other year data as TST, the prediction accuracies estimated from the SM were similar to those estimated from the MM, despite in the within location analyses or in the across location analyses. However, the prediction accuracies estimated from the across location analyses were higher than those estimated from the within location analyses, which indicated the importance of the multiple-environment trials. Moreover, the prediction accuracies estimated from the 2 years of data used as TRN were higher than those estimated from the 1 year of data used as TRN, which indicated the effect of increasing TRN size on prediction accuracy improvement.

The prediction accuracies estimated from the independent validation schemes are shown in Table 3, when the size of the TRN using 2 years of breeding data was adjusted the same as the size of the TRN using 1 year of data, and the third year’s data were used as the TST set. In the within location analyses, the prediction accuracies of SM had an average value of 0.25 and ranged from 0.14 to 0.37. In the across location analyses, the prediction accuracies of SM had an average value of 0.35 and ranged from 0.26 to 0.46, and the prediction accuracies estimated from the across location analyses were higher than those estimated from the within location analyses, indicating the importance of the multiple-environment trials for improving the prediction accuracy.

**Table 3:** The prediction accuracies and the standard errors of prediction accuracies were estimated from the independent validation schemes, when the size of the TRN using 2 years of breeding data was adjusted the same as the size of the TRN using 1 year of data, and the third year’s data were used as TST set. The random sampling selection was repeated 30 times. SM was applied in the within and across location analyses

Location	Year in TST	*Adjusted size of TRN same as below year		
		2015	2016	2017
AF	^+^2015		0.37 (0.05)	0.31 (0.05)
	^+^2016	0.19 (0.04)		0.14 (0.03)
	^+^2017	0.29 (0.04)	0.32 (0.02)	
TL	^+^2015		0.22 (0.04)	0.18 (0.05)
	^+^2016	0.19 (0.00)		0.28 (0.02)
	^+^2017	0.21 (0.02)	0.26 (0.01)	
Across	^+^2015		0.38 (0.04)	0.46 (0.06)
Locations	^+^2016	0.26 (0.03)		0.33 (0.02)
	^+^2017	0.31 (0.03)	0.37 (0.02)	

Standard errors of prediction accuracies from 30 replications are shown between brackets.

*Adjusted size of the TRN using 2 years of breeding data was the same as the size of below single year’s breeding data

^+^The TST of 2015 breeding data was predicted with the adjusted size of the 2016 and 2017 breeding data, the TST of 2016 breeding data was predicted with the adjusted size of the 2015 and 2017 breeding data, and the TST of 2017 breeding data was predicted with the adjusted size of the 2015 and 2016 breeding data

In both the within and across location analyses, the prediction accuracies estimated from the adjusted size of 2 years of data used as TRN were similar to those estimated from the 1 year of data used as TRN, while the prediction accuracies estimated from the adjusted size of 2 years of data used as TRN were lower than those estimated from the 2 years of data used as TRN. This result confirmed that the improved prediction accuracies estimated from the 2 years of data as TRN are mainly caused by larger TRN size, rather than by incorporating environmental variations.

## Strengthening the relationship between TRN and TST, and increasing the TRN size to improve the prediction accuracies estimated from the independent validation schemes

**Fig. 3:**
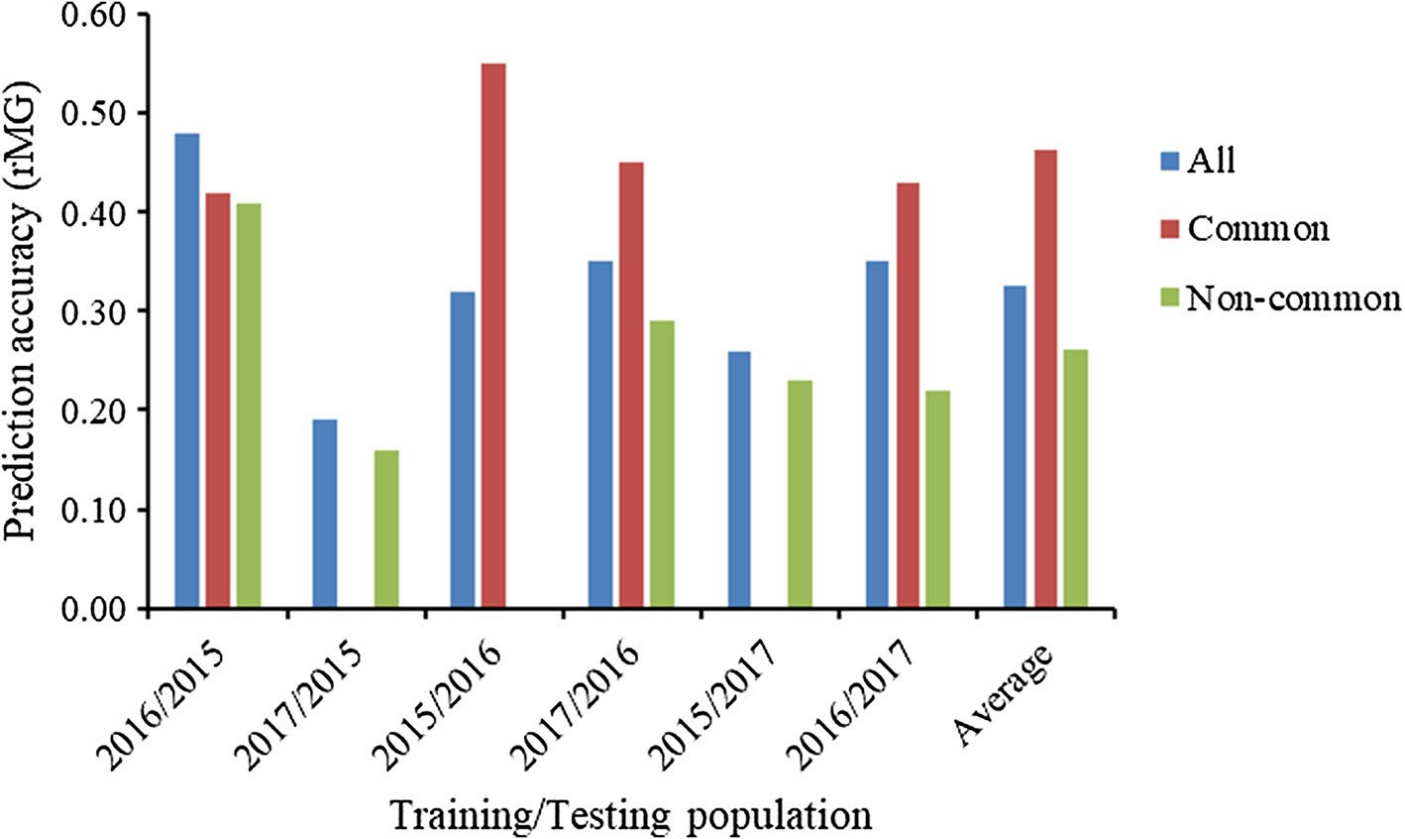
The prediction accuracies estimated from the independent validation schemes; when the single-environment model (SM) was applied in the across location analyses, the across years’ predictions were implemented by using TRN and TST from (1) all the DH lines from different years (all blue bars); (2) the full-sib- or half-sib-related DH lines shared between the different years (common red bars); and (3) the DH lines without any shared parental lines between the different years (non-common green bars)

The prediction accuracies estimated from the independent validation schemes are shown in Fig. 3; when the SM was applied in the across location analyses, the across years’ predictions were implemented by using TRN and TST from (1) all the DH lines from different years (the blue bars in Fig. 3 represent the same values with those values of the across location analyses in Table 2 estimated from the SM); (2) the full-sib- or half-sib-related DH lines shared between the different years; and (3) the DH lines without any shared parental lines between the different years. Only a few full-sib- or half-sib-related DH lines are shared between 2015 and 2017, so the across years’ predictions between 2015 and 2017 were not implemented with the shared full-sib- or half-sib-related DH lines.

The across years’ prediction accuracies estimated with the shared full-sib- or half-sib-related DH lines were higher than those estimated with all the DH lines from different years, except for using the 2016 breeding data as TRN to predict the 2015 breeding data as TST. The lowest prediction accuracies were observed, when the across years’ predictions were made with the DH lines without any shared parental lines between the different years. These results indicated that the prediction accuracies estimated from the across years’ predictions could be improved by strengthening the relationship between TRN and TST and incorporating the shared full-sib- or half-sib-related DH lines as TRN and TST.

In addition to strengthening the relationship between TRN and TST, the prediction accuracies estimated from the across years’ predictions also could be improved by increasing the size of TRN (Table 4). When the 2 years of data were used as TRN to predict the other year’s data as TST, the prediction accuracies of SM had an average value of 0.41 and ranged from 0.36 to 0.50 in the across location analyses. The prediction accuracies were further improved by converting 50% of the TST to TRN. The accuracies of predicting 50% of the 2017 breeding data, 50% of the 2016 breeding data, and 50% of the 2015 breeding data increased to 0.50, 0.56, and 0.50, when the TRN was formed by 2 years of breeding data with 50% of the 2017 breeding data, 50% of the 2016 breeding data, and 50% of the 2015 breeding data, respectively.

**Table 4:** The prediction accuracies estimated from the independent validation schemes using 2 years of data as training population, or using training population consisted of 2 years of breeding data and 50% of third year’s data converted from testing population to training population, when the single-environment model (SM) was applied in the across location analyses

Training population	Testing population	Prediction accuracy
2015 + 2016	2017	0.38
2015 + 2017	2016	0.36
2016 + 2017	2015	0.50
2015 + 2016 + 50% 2017	50% 2017	0.50 (0.03)
2015 + 2017 + 50% 2016	50% 2016	0.56 (0.02)
2016 + 2017 + 50% 2015	50% 2015	0.50 (0.04)

Standard errors of prediction accuracies from 50 replications are shown between brackets

## Discussion

As part of the routine maize product development pipeline at CIMMYT, thousands of DH lines are able to be generated for each breeder every year, the main task for a maize breeder is to estimate the breeding values of thousands of DH lines, rather than to generate thousands of homozygous inbred lines annually. However, it is very difficult to phenotype thousands of newly developed DH lines in the first-stage yield testing in multi-environment trials and advance them to second-stage yield testing trials based on their phenotype-based breeding values, due to the limited space and resources for phenotyping. In the present study, a total of 1528 DH lines, phenotyped in multiple-environment trials in three consecutive years and genotyped with a low-cost per-sample genotyping platform of rAmpSeq, were used to explore how to implement GS to improve breeding efficiency in a maize doubled haploid breeding program. rAmpSeq is a newly developed sequencing method; the genotyping cost is less than 5 US dollars per sample (Buckler et al. 2016), which is cheaper than the phenotyping cost of a single plot evaluated at CIMMYT maize breeding program. The average prediction accuracy estimated from the fivefold cross-validation schemes was above 0.60 across all the scenarios, which are consistent with several previous maize studies (Crossa et al. 2014; Zhang et al. 2015). These results indicated that a low-cost per-sample genotyping platform of rAmpSeq offers the opportunity of implementing GS to replace the expensive multiple-environment phenotyping trials, to reduce the breeding cost of the first-stage yield testing, and to predict the GEBVs of the un-phenotyped DH lines for further selection. However, the prediction accuracies estimated from the fivefold cross-validation schemes are always higher than the prediction accuracies estimated from a real maize breeding program, because the breeders always prefer to phenotype as less breeding materials as they can, when they build the TRN. Recently, the maize breeding program of CIMMYT in Kenya validated that the GS performed similarly as the phenotypic selection in the first-stage yield testing, when the testcrosses of 50% new developed DH lines were evaluated as TRN to predict the GEBVs of the remaining 50% un-phenotyped DH lines for further selection. Moreover, the GS reduced the cost by 32% over the PS with similar selection gains (Beyene et al. 2019).

Instead of implementing GS to predict the remaining 50% un-phenotyped DH lines, the 100% un-phenotyped DH lines also could be predicted by using the historical breeding data as TRN; this requires to build a multiple-year TRN set. In the present study, a total of 1528 DH lines, phenotyped in three consecutive years, were used to estimate the across years’ genomic prediction accuracies, when the TRN and TST were from different years. Results of the present study showed that the average prediction accuracies of MM were 0.23 in the within location analyses and 0.32 in the across location analyses, when the 1-year data were used as TRN to predict the other year data as TST. When the 2 years of data were used as TRN to predict the other year data as TST, the average prediction accuracies of MM increased to 0.34 in the within location analyses and 0.42 in the across location analyses; these results indicated that the prediction accuracy of GS needs to be improved by incorporating the historical breeding data from multiple years as TRN. When multiple years of historical breeding data are used as TRN, the larger size of TRN contributes to the improvement in the prediction accuracy. The development of a multiple-year TRN set will allow GS advancing all the 100% un-phenotyped DH lines directly to the second-stage yield testing trials, and the first-stage yield testing is skipped. No phenotyping cost occurs in the first-stage selection; the selection is only based on predictions. Compared with the breeding strategy implemented by Beyene et al. (2019), the total breeding cost of the breeding strategy proposed in the present study is further reduced; it will reduce the cost by more than 32% over the PS. This breeding strategy significantly saves the cost of the multiple-environment trials in the first-stage yield testing, as well as saving the time of testcross formation and evaluation of the first-stage yield testing (Beyene et al. 2019). These independent validation schemes mimic real maize breeding situations. The results of this study also showed that the prediction accuracies estimated from the independent validation schemes could be further improved by strengthening the relationship between TRN and TST. When the 1-year data were used as TRN to predict the other year data as TST, the prediction accuracy estimated with the shared full-sib- or half-sib-related DH lines was higher than that estimated with all the DH lines, and the prediction accuracy estimated with DH lines without any shared parental lines between the different years was lower than that estimated with all the DH lines. These results agree with the observations of Brandariz and Bernardo (2019); the prediction accuracy could be improved by strengthening the relationship between TRN and TST. The prediction accuracies were increased from ~ 0.41 to ~ 0.50, when the TRN consisted of 2 years of breeding data and 50% of third year’s data converted from TST to TRN. These results show that TRN set using the historical breeding data from multiple years and adding more TRN materials with closer relationship with TST set could improve the prediction accuracy, when it is used to predict the similar germplasm untested in any environment.

Multiple-environment trials play an important role in early-stage testcross testing. Several studies showed that incorporating genotype by environment interaction into the statistical models is able to improve the genomic prediction accuracy (Burgueño et al. 2012; Jarquín et al. 2014; Sousa et al. 2017; Zhang et al. 2015). In this study, the MM outperformed the SM in the fivefold cross-validation schemes on improving the prediction accuracy, and the prediction accuracies estimated from the across location analyses were consistently higher than those estimated from the within location analyses. This result indicated the importance of conducting multiple-environment trials in early-stage testcross testing and incorporating genotype by environment interaction into the genomic prediction model. However, MM had a similar performance as SM in all the independent validation schemes; the development of advanced models incorporating genotype by environment interaction still demands to improve prediction accuracy.

The prediction accuracy can be increased by modeling the tester effect into the prediction model, it had been discussed in several previous studies, and it is more important in genomic prediction of the hybrid performance (Albrecht et al. 2011 and 2014). In the present study, 11 testers were used for making testcrosses for evaluation of the 19 multiple location trials conducted in 3 years, and only one tester overleaped between years. In the early-stage testcross testing, a large number of tested inbred lines are derived from genetically diverse parents and populations; the main objective of using multiple testers are to evaluate the general combing ability and breeding value of this large number of tested inbred lines, rather than to predict the best performance hybrid made between the tested inbred line with a specific tester. Therefore, we did not incorporate the tester effect into the prediction model in the present study, which will be assessed in further studies.
